# Different Associations between *DC-SIGN* Promoter-336G/A (*rs4804803*) Polymorphism with Severe Dengue in Asians and South-Central Americans: a Meta-Analysis

**DOI:** 10.3390/ijerph16081475

**Published:** 2019-04-25

**Authors:** Jiangping Ren, Zhengting Wang, Enfu Chen

**Affiliations:** 1 Zhejiang Provincial Centre for Disease Control and Prevention, Hangzhou 310051, China; jpren@cdc.zj.cn (J.R.); ztwang@cdc.zj.cn (Z.W.); 2 Key Laboratory of Vaccine, Prevention and Control of Infectious Disease of Zhejiang Province, Hangzhou 310051, China; 3 Field Epidemiology Training Program of Zhejiang Province, Hangzhou 310051, China

**Keywords:** dengue, polymorphism, *DC-SIGN*, *rs4804803*, Meta-analysis

## Abstract

*Objective*: This study was conducted to identify the association between *rs4804803* polymorphism in *DC-SIGN* with the susceptibility of severe dengue. *Methods*: A comprehensive search was conducted to identify all eligible papers in PubMed, Web of Science, China National Knowledge Infrastructure (CNKI), and Google Scholar. Odds ratios (ORs) and corresponding 95% confidence intervals (95% CIs) were used to assess the association. Subgroup analyses were performed by ethnicity. Sensitivity analyses were performed through employing different statistical models (fixed versus random effect model). *Results*: A total of nine papers and 12 studies, with 1520 severe dengue and 1496 clinical dengue infection were included. The overall meta-analysis revealed significant associations between rs4804803 and severe dengue under the recession (*GG* versus *GA/AA*: OR = 0.44, 95%CI, 0.23–0.82) and a codominant model (*GG* versus *AA*: OR = 0.43, 95%CI, 0.23–0.81), but sensitivity analysis indicated that the significant pooled ORs were not robust. The subgroup analysis suggested that the carrier of G in *rs4804803* was a risk factor for severe dengue under dominant (*GG/GA* versus *AA*: OR = 1.86,95%CI, 1.01–3.45), superdominant (*GA* versus *GG/AA*: OR = 1.81,95%CI, 1.02–3.21) and a codominant (*GA* versus *AA*: OR=1.82,95%CI, 1.02–3.26) models in Asians, while it was a protective factor for severe dengue in South-central Americans under recessive (*GG* versus *GA/AA*: OR = 0.27,95%CI, 0.10–0.70) and codominant (*GG* versus *AA*: OR=0.24,95%CI, 0.09–0.64) models. The results from subgroup analysis were robust. *Conclusions*: Dendritic cell-specific intercellular adhesion molecule-3-grabbing non-integrin (*DC-SIGN*) promoter-336G/A (*rs4804803*) polymorphism is association with severe dengue, and it acts in different directions for Asians and South-central Americans.

## 1. Introduction

Dengue is the most prevalent mosquito-borne infectious disease, which is endemic more than 125 countries around the world, especially South-East Asia, the Americas, the Western Pacific and Africa. In the past 50 years, the incidence has increased 30 fold and the affected area has extended to new countries and from urban to rural settings [1]. It is estimated that about half of the world’s population is now at risk. The most recently published study about the global burden of dengue indicated that there were 58.4 million (23.6 million–121.9 million) apparent cases with incidence of 810·1 (327.4–1690.8) per 100,000 and 9110 (5630–10,842) deaths with mortality of 1.27 (0.79–1.52) per million in 2013, and dengue was responsible for 1.14 million (0.73–1.98 million) disability-adjusted life-years [2]. The driving forces behind the emergence and reemergence include climate change, poorly planned urbanization, globalization, viral evolution and adaption, and deficiencies in water supply and garbage disposal [3]. Dengue virus (DENV) is a member of the family Flaviviridae under the genus *Flavivirus*, which also includes Zika virus, Yellow Fever virus and West Nile virus.It is antigenically divided into four serotypes: DENV-1, DENV-2, DENV-3, and DENV-4. The clinical manifestations of DENV infection is protean, from asymptomatic infection, mildly symptomatic disease, to life-threatening severe dengue, including dengue hemorrhagic fever (DHF) and dengue shock syndrome (DSS).

According to the World Health Organization (WHO, 2009) dengue case classification, the criteria for severe dengue include severe plasma leakage leading to shock or fluid accumulation with respiratory distress, severe bleeding as evaluated by a clinician, or severe organ impairment involving liver (AST or ALT ≥1000), central nervous system (impaired consciousness), heart and other organs [1,4].The identified risk factors for severe dengue include second infection, age, delay in admission, pregnancy, infection with DENV-2 and certain chronic diseases (such as diabetes, cardiac disorders, asthma) [4,5,6,7,8,9,10,11,12,13,14]. The host-specific genetics is also a contributing factor [15]. Studies indicated that African ancestry was a protective factor for severe dengue [4,16,17]. Patients with AB blood group had higher risk of developing DHF [5]. A large number of association studies between genetic polymorphisms and the severity of dengue have been conducted in the past 20 years. Dendritic cell-specific intercellular adhesion molecule-3-grabbing non-integrin (DC-SIGN, also known as CD209) is a type II C-type lectin, which plays an important role in the early interaction of a pathogen with dendritic cell (DC), DC–T cell interaction, DC migration and pathogen uptake [18,19,20]. Other than being involved in viral entry, studies indicated that it also has a role in the progression to severe forms of disease during dengue infection [21,22,23]. *rs4804803*, a single nucleotide polymorphism (SNP) in the promoter of *DC-SIGN*, is one of the widely studied loci in the research about the genetic susceptibility of dengue and severe dengue, but the results are quite controversial [19,24,25,26,27,28,29,30,31]. Meta-analysis is the quantitative, scientific synthesis of research results and can establish evidence-based practice and resolve seemingly contradictory research outcomes [32]. So, we performed this meta-analysis to identify the association of *rs4804803* with the severity of dengue.

## 2. Materials and Methods

### 2.1. Search Strategy and Inclusion Criteria

Electronic searches were conducted in PubMed, Web of Science, China National Knowledge Infrastructure (CNKI) and Google Scholar with the keywords of “dengue” and “*CD209* or *DC-SIGN*” to identify relevant articles published up until 31 December 2018. The references of related articles were also screened to identify papers.

**Figure 1:**
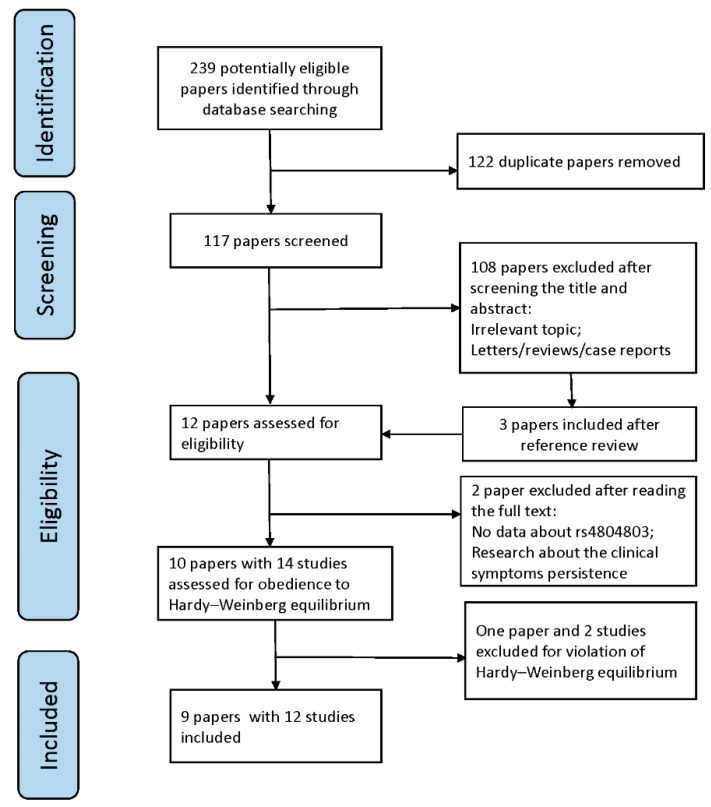
PRISMA flow diagram of systematic search results and study selection.

All the studies included in this meta-analysis met the following criteria: (1) a human study with full paper available; (2) a case-control study on the association of *rs4804803* polymorphism in *DC-SIGN* with the development of severe dengue in clinical dengue infection; (3) the original data about the genotype distribution is accessible; (4) published studies;(5) the distributions of genotypes did not differ significantly from the Hardy–Weinberg equilibrium (HWE). Case reports, reviews or articles without full text were excluded. Duplicated studies were carefully screened, then those with bigger sample size, higher quality or published most recently were kept. PRISMA guidelines were followed in the study selection (Figure 1).

### 2.2. Data Extraction

The following information was extracted in each study included: the first author, year of publication, country, WHO criteria for dengue case classification, ethnicity, matching criteria of controls, genotype method, sample size, the numbers of the severe dengue and uncomplicated clinical dengue case (indicated as DF in this paper) with different genotypes.

### 2.3. Quality Assessment

The quality of each study was assessed independently by two researchers according to the Newcastle–Ottawa Scale (NOS) for case-control study. This criteria include three sectors: selection (a maximum score of four), comparability (a maximum score of two) and exposure (a maximum score of three). The third researcher was asked to conduct the quality assessment if inconsistent existed.

### 2.4. Statistical Analysis

The deviation from HWE was examined in both severe dengue and DF group with a web tool [33]. Pooled odds ratio (OR) with 95% confidence interval (CI) and *z* test (*p* < 0.05 was considered as significant difference) were used to measure the association between *rs4804803* polymorphism and the risk of severe dengue under four inheritance models (dominant: *GG/GA* versus *AA*; recessive: *GG* versus *GA/AA*; codominant: *GG* versus *AA*, *GA* versus *AA*; superdominant: *GA* versus *GG/AA*), respectively. *I^2^* statistic was used to measure the magnitude of heterogeneity and *Q* test was used to identify whether the heterogeneity was statistical significantly. Fixed effect model was employed in meta-analysis when *p* value of *Q* test was more than 0.10 or *I^2^* value was less 50%, otherwise random effect model would be adopted. Pooled OR and its 95%CI were obtained by using the Mantel–Haenszel method in the fixed effect model or by using the Der Simonian and Laird method in the random effect model. When heterogeneity was statistically significantly, meta-regression was conducted to explore the possible source. A funnel plot, Egger’s regression test and Begg’s test were used to identify whether publish bias existed, and *p* < 0.05 was regarded as statistically significant. Sensitivity analysis was performed through employing different statistical models (fixed effect model versus random effect model). The pooled OR was robust if the result from the fixed and random effect model was consistent. All of the analyses were conducted with Stata 10.0 software (StataCorp, College Station, TS, USA), and two-sided tests with *p* value less than 0.05 was considered statistically significant unless there was specification.

## 3. Results

### 3.1. Study Inclusion and Characteristics

A total of 239 papers were identified through database searching, and 122 of them were excluded for duplication (Figure 1). After reviewing the references and roughly screening the title and abstract, 12 papers were included. The full text of the included papers were accessible. Two papers were removed after reading: one paper had no data about *rs4804803* [34]; another paper was a study about the persistence of dengue clinical symptoms [35]. Then, 10 papers with 14 studies were tested for the obedience to HWE, and one paper and two studies were excluded for their violation. Finally, nine papers and 12 studies, with 1520 severe dengue and 1496 DF cases, were included (Table 1). All the papers in this meta-analysis were published during 2005 to 2018, and patients were from Asia or South-central America (SCA).

### 3.2. Quantitative Data Synthesis

**Figure 2:**
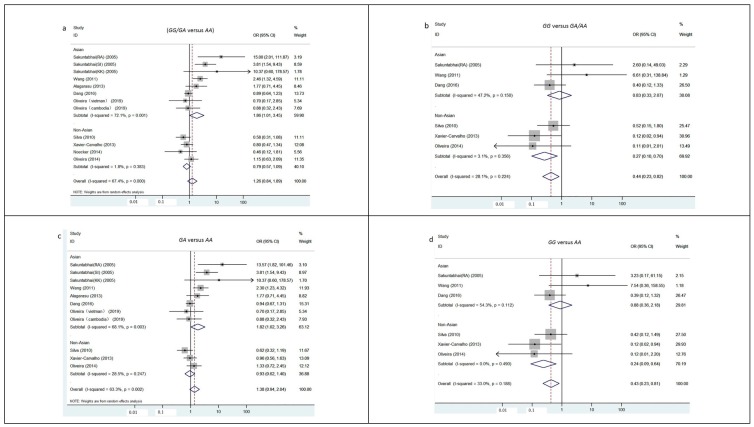

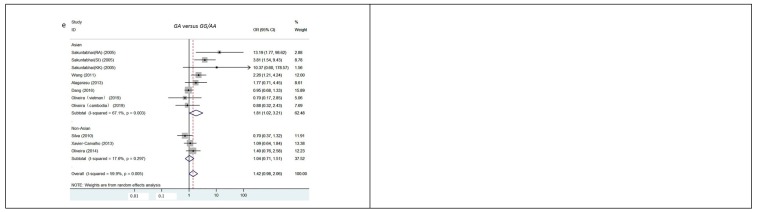
Subgroup meta-analysis by ethnicity on *rs4804803* polymorphism and severity of dengue. Five studies, which were Sakuntabhai(SI) and Sakuntabhai(KK) published in 2005, Alagarasu published in 2013, Oliveira(vietman) and Oliveira(Cambodia) published in 2018, were automatically excluded in genotype analysis of GG versus GA/AA and GG versus AA due to absence of GG individuals in both groups; Other than the analysis under dominant inheritance model, the study conducted by Noecker et al. was automatically excluded in all the genotype analysis due to no information about the distribution of GG and GA genotype obtained. (a) Subgroup meta-analysis under dominant inheritance model (*GG/GA* versus *AA*). (b) Subgroup meta-analysis under recessive inheritance model (*GG* versus *GA/AA*). (c) Subgroup meta-analysis under codominant inheritance model (*GA* versus *AA*). (d) Subgroup meta-analysis under codominant inheritance model (*GG* versus *AA*). (e) Subgroup meta-analysis under superdominant inheritance model(*GA* versus *GG/AA*).

The results from the pooled meta-analyses showed that, except under the recession and a codominant model, no significant associations were found under any other inheritance models (*GG/GA* versus *AA*: OR = 1.26, 95%CI, 0.84–1.19; *GA* versus *AA*: OR = 1.38, 95%CI, 0.94–2.04; *GA* versus *GG/AA*: OR = 1.42, 95%CI, 0.98–2.06, Figure 2). The *G* allele of *rs4804803* in DC-SIGN reduced the severity of clinical dengue infections under the recession (*GG* versus *GA/AA*: OR = 0.44, 95%CI, 0.23–0.82) and a codominant model (*GG* versus *AA*: OR = 0.43, 95%CI, 0.23–0.81). The results from subgroup meta-analysis uncovered the different role of *rs4804803* in the development of severe dengue for different ethnicity. The carrying of G in *rs4804803* acted as a risk factor when progression to severe dengue under dominant (*GG/GA* versus *AA*: OR = 1.86, 95%CI, 1.01–3.45), superdominant (*GA* versus *GG/AA*: OR = 1.81, 95%CI, 1.02–3.21) and a codominant (*GA* versus *AA*: OR = 1.82, 95%CI, 1.02–3.26) model in Asians, while it reduced the risk of severe dengue in SCA under recessive (*GG* versus *GA/AA*: OR = 0.27, 95%CI, 0.10–0.70) and a codominant (*GG* versus *AA*: OR = 0.24, 95%CI, 0.09–0.64) models (Figure 2).

### 3.3. Heterogeneity and Publication Bias

The result from the *Q* test and *I^2^* test implied that there were significant heterogeneities among studies under dominant (*GG/GA* versus *AA*: *I^2^* = 67.4%, *p* < 0.001), superdominant (*GA* versus *GG/AA*: *I^2^* = 59.9%, *p* = 0.005) and codominant (*GA* versus *AA*: *I^2^* = 63.3%, *p* = 0.002) models. To explore the source of heterogeneities, meta-regression analyses by ethnicity and matching criteria of controls were conducted. The result suggested that ethnicity might be one of the reasons for heterogeneity under the dominant model (*p* = 0.044, Table 2).

**Figure 3:**
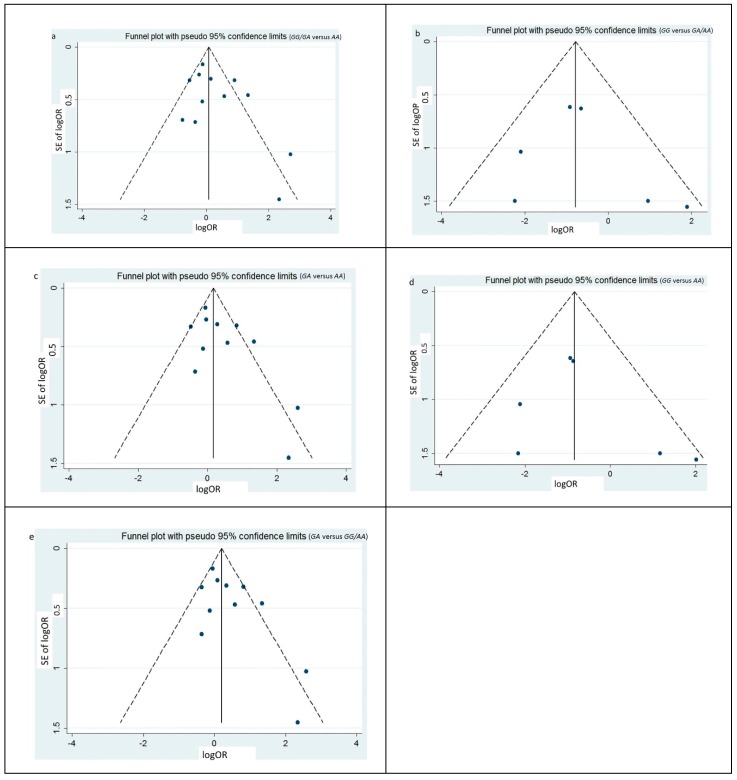
Funnel plots for publication bias of the meta-analysis on *rs4804803* polymorphism and severity of dengue. Data from all the eligible studies, both from Asians and SCA, were included. (a) Funnel plots for publication bias under dominant inheritance model (*GG/GA* versus *AA*). (b) Funnel plots for publication bias under recessive inheritance model (*GG* versus *GA/AA*). (c) Funnel plots for publication bias under codominant inheritance model (*GA* versus *AA*). (d) Funnel plots for publication bias under codominant inheritance model (*GG* versus *AA*). (e) Funnel plots for publication bias under superdominant inheritance model (*GA* versus *GG/AA*).

The shape of the funnel plots under four models were symmetrical, which suggested no publication bias existing (Figure 3). The result from Egger’s regression test and Begg’s test confirmed that there was no publication bias (*p* < 0.05, Table 3).

### 3.4. Sensitivity Analysis

The sensitivity analysis showed that, except under dominant model, the pooled ORs and 95% CIs were inconsistent between the fixed effect model and random effect model under any other inheritance models (Table 4). It indicated that the significant pooled ORs were not robust under those three models. In consideration of the possible different role of *rs4804803* during the development of severe dengue in Asian and SCA, and ethnicity recognized as a possible source of heterogeneity under the dominant model, subgroup sensitivity analyses by ethnicity were conducted. The ORs and 95% CIs were consistent between the fixed effect model and random effect model under the four inheritance models in Asians and SCA, respectively, which indicated that the results from the subgroup meta-analysis were robust (Table 4).

## 4. Discussion

DC-SIGN is preferentially expressed on myeloid DCs [18]. It is composed of 404 amino acids and four domains: a cytoplasmic domain, a transmembrane domain, an extracellular neck domain, and a carbohydrate recognition domain (CRD) [18,20]. The cytoplasmic domain is responsible for receptor signaling for the binding, phagocytosis and intra-cellular trafficking of ligand molecules. The transmembrane domain, consisting of 15 amino acids, anchors the proteins to the cytoplasmic membrane. The neck domains, completed of seven complete repeats and one incomplete repeat, stabilizes the tetramer of the extra-cellular portion, enhances the affinity between DC-SIGN and its ligand and amplifies the signal. The CRD in DC-SIGN is Ca^2+^ dependent and can selectively recognize and bind to high-mannose oligosaccharides. Generally, DC-SIGN performs cell-adhesion and pathogen recognition functions [18]. The *DC-SIGN* gene is located on chromosome 19p13.2-3. *rs4804803*, a SNP in the promoter of *DC-SIGN*, is one of the most frequently studied genetic locus. In vitro, *rs4804803* affected a Sp1 binding site and the level of transcription of DC-SIGN, with lower DC-SIGN expression for G allele in *rs4804803* [30]. The population-based genetic association studies indicated that the polymorphism of *rs4804803* was related to the susceptibility of HIV-1, tuberculosis, Chikungunya [38,39,40,41].

Naturally, DENV is introduced into human skin by an infected mosquito vector. Its primary target cells in the skin are Langerhans cells or immature DCs. Studies indicated that DC-SIGN not only acts as an attachment factor during DENV infection but also plays a role in virus entry [42]. Immature DENV, non-infectious without antibodies, could infect immature DCs through interaction with DC-SIGN. It suggested that immature DENV particles could contribute to dengue pathogenesis through DC-SIGN during primary infection [43]. Although a study indicated that a carrier of the G allele in rs4804803 expressed lower level of DC-SIGN in vitro, monocyte-derived DCs from individuals with *AG* genotype in *rs4804803* had a higher DC-SIGN expression than those with the AA genotype in response to dengue infection, and higher TNF-a, IL-12p40, and IP-10 production, lower viral replication were also noted [19]. The reason for this contradiction should be explored further. The genetic association studies showed that the polymorphism of *rs4804803* might be related to the susceptibility of dengue, but it remains controversial. The studies conducted in the Taiwan and Mexican populations indicated that the *G* allele in *rs4804803* increased the risk of clinical dengue infections, but a studies from Thailand population implied it as a protective factor [19,25,30]. No association was found in studies from Brazil and Western India [26,27].

Recently, studies suggested that the DC-SIGN might also have a role in the progression to severe dengue. Antibody-dependent enhancement (ADE) is the phenomenon that heterotypic antibodies do not neutralize virions of the subsequent infecting DENV type, but facilitate virus entry, replication in target immune cells, and consequently lead to higher viremia [44]. It is widely regarded as one of the reason for the development of severe dengue [45]. A study indicated that ADE was inversely correlated with surface expression of DC-SIGN in vitro: Mature DC, which expresses lower level of DC-SIGN, exhibited ADE, while immature DC, expressing higher levels of DC-SIGN, did not undergo ADE [21]. Platelets from DENV-infected patients present signs of activation, mitochondrial dysfunction, and activation of apoptosis caspase cascade, which may contribute to the genesis of thrombocytopenia. DC-SIGN, as a critical receptor, was involved in this progress of DENV-dependent platelet activation [22,23]. Expression of DC-SIGN was downregulated on platelets in patients with dengue infection, and the decreased receptor expression diminishes platelet activation [46]. Inconsistency was also noted when researchers tried to explore the role of *rs4804803* in the development of severe dengue. *GG* genotype of *rs4804803* was recognized protectively to severe dengue in a studies from Brazil [28], but higher risk was found from a study in Thailand and in Taiwan, China, respectively [19,30]. No associations were recognized in studies from Mexican, Western India, and another two studies from Brazil and Thailand [25,26,27,29,36].

The overall meta-analyses showed that the carrier of *G* in *rs4814803* was a significant protector for severe dengue under the recession (*GG* versus *GA/AA*, OR < 1) and a codominant model (*GG* versus *AA*, OR < 1). However the significant pooled ORs were not robust and the heterogeneities between different studies were noted under four inheritance models. Ethnicity was identified as a possible source of heterogeneity. The further subgroup meta-analysis indicated that *rs4804803* might play a different role in progression to severe dengue in different ethnicities. The carrier of *G* in *rs4804803* acted as a risk factor for the development of severe dengue under dominant (*GA/GG* versus *AA*: OR > 1), superdominant (*GA* versus *AA/GG*: OR > 1) and a codominant (*GA* versus *AA*: OR > 1) models in Asians, while it reduced the risk in SCA under recessive (*GG* versus *GA/AA*: OR < 1) and a codominant (*GG* versus *AA*: OR < 1) model. The results from subgroup analysis were robust according the sensitivity analysis. The meta-analysis conducted by Xavier-Carvalho et al. [28] also indicated the different association of *rs4804803* with severe dengue: the *G* allele of *rs4804803* was associated with higher risk of severe dengue in Asians, but was a protective factor in Brazilians. Another study from Pabalan et al. [24] also suggested it is a protective factor for DHF in the SCA population when compared with healthy and asymptomatic control, but no association was conferred in Asians. Studies conducted in Brazil indicated that the G carrier was a protective factor for the symptoms of headache and arthralgia in dengue fever [26]. Besides dengue, the polymorphism of *rs4804803* was also associated with the severity of tuberculosis, severe acute respiratory syndromes and tick-borne encephalitis [37,38,47]. The opposite effect of *rs4804803* on the development of severe dengue is not inexplicable in consideration the role of DC- SIGN in ADE and DENV-dependent platelet activation: the expression of DC-SIGN was inversely correlated with ADE, but positively correlated with DENV-dependent platelet activation [21,22,46]. The other possible reasons for the different role of *rs4804803* might be related to: the different frequency of G allele in rs4804803 between ethnicities; the different distribution of the serotype and the ratio of secondary globally; other factors, including genetic factors, that might determine the different role of *rs4804803* in *DC-SIGN* during dengue infection.

## 5. Conclusions

In summary, the *G* carrier of *rs4804803* in *DC-SIGN* has a higher risk for progression to severe dengue in Asians and a lower risk in SCA. Regarding the multiple factor for the development to severe dengue, there are limitations in this meta-analysis. Globally, the epidemic status of dengue across the whole world and in different years are different, so the ratio of secondary infection, one of the important reasons for severe dengue, might be inconsistent between studies. However, this was not included in the meta-analysis. Information on serotypes was not available from all of the included papers, so this was also not included in the analysis.

## Figures and Tables

**Table 1:** Characteristics of the individual studies in this meta-analysis.

First Author	Publish Year	Ethnicity	Country	WHO Criteria	Matching Criteria of Controls	Method	*N*		Score NOS
							Severe Dengue	DF	
Sakuntabhai [30]	2005	Asian	Thailand (RA)	1997	Gender	TaqMan	144	50	6
Sakuntabhai [30]	2005	Asian	Thailand (SI)	1997	Gender	TaqMan	124	66	6
Sakuntabhai [30]	2005	Asian	Thailand (KK)	1997	Gender	TaqMan	87	27	6
Silva [29]	2010	SCA	Brazil	1997	Gender and age	BeadArray	31	128	9
Wang [19]	2011	Asian	China	1997	Gender	TaqMan	106	157	6
Xavier-Carvalho [28]	2013	SCA	Brazil	2009	Age	TaqMan.	52	122	6
Alagarasu [27]	2013	Asian	India	1999	Unmentioned	PCR-RFLP	19	64	5
Noecker [25]	2014	SCA	Mexican	2009	Gender	TaqMan	33	51	6
Oliveira [26]	2014	SCA	Brazil	2009	Gender and age	TaqMan	67	74	8
Dang [36]	2016	Asian	Thailand	1997	Gender	TaqMan	415	331	6
Oliveira [37]	2018	Asian	Vietnam	1997	Unmentioned	TaqMan	20	21	6
Oliveira [37]	2018	Asian	Cambodia	1997	Unmentioned	TaqMan	93	57	6

SCA, South-central American; PCR-RFLP, polymerase chain reaction and restriction fragment length polymorphism.

**Table 2:** Meta-regression by ethnicity and matching criteria of controls under different inheritance models.

Model of inheritance		Factor	*Β* (95%CI)	*t*	*p*
Dominant	*GG/GA* versus *AA*	Matching criteria of controls	0.53 (−0.44, 1.49)	1.23	0.249
		Ethnicity	1.35 (0.04, 2.66)	2.33	0.044
Recessive	*GG* versus *GA/AA*	Matching criteria of controls	0.98 (−4.41, 6.37)	0.58	0.603
		Ethnicity	2.20 (−3.00, 7.39)	1.35	0.271
Codominant	*GG* versus *AA*	Matching criteria of controls	0.86 (−4.69, 6.40)	0.49	0.657
		Ethnicity	2.31 (−3.02, 7.65)	1.38	0.262
	*GA* versus *AA*	Matching criteria of controls	0.47 (−0.60, 1.55)	1.02	0.338
		Ethnicity	1.13 (−0.39, 2.66)	1.71	0.125
Superdominant	*GA* versus *GG/AA*	Matching criteria of controls	0.44 (−0.61, 1.50)	0.97	0.359
		Ethnicity	0.99 (−0.50, 2.49)	1.53	0.165

**Table 3:** Test of publication bias under different inheritance models.

Model of inheritance			Egger’s			Begg’s	
			*t*	*p*		*z*	*p*
Dominant	*GG/GA* versus *AA*		1.56	0.150		1.17	0.244
Recessive	*GG* versus *GA/AA*		0.54	0.616		1.13	0.260
Codominant	*GG* versus *AA*		0.74	0.499		0.75	0.452
	*GA* versus *AA*		1.96	0.081		1.25	0.213
Superdominant	*GA* versus *GG/AA*		2.05	0.071		1.09	0.276

**Table 4:** Sensitivity analysis under different inheritance models through adopting different effect models.

Model of inheritance		Group	*I^2^* (%)	Fixed effect model				Random effect model		
				*z*	*p*	OR(95%CI)		*z*	*p*	OR(95%CI)
Dominant	*GG /GA* versus *AA*	Overall	67.4	1.67	0.096	1.17 (0.97, 1.42)		1.12	0.263	1.26 (0.84, 1.89)
		Asian	72.1	3.12	0.002	1.46 (1.15, 1.85)		1.98	0.048	1.86 (1.01, 3.45)
		SCA	1.8	1.48	0.140	0.79 (0.57, 1.08)		1.43	0.153	0.79 (0.57, 1.09)
Recessive	*GG* versus *GA/AA*	Overall	28.1	2.57	0.010	0.44 (0.23, 0.82)		1.59	0.112	0.47 (0.19, 1.19)
		Asian	47.2	0.40	0.691	0.83 (0.33, 2.07)		0.21	0.834	1.22 (0.20, 7.51)
		SCA	3.1	2.67	0.008	0.27 (0.10, 0.70)		2.29	0.022	0.30 (0.11, 0.84)
Codominant	*GG* versus *AA*	Overall	33.0	2.63	0.009	0.43 (0.23, 0.81)		1.54	0.122	0.46 (0.18, 1.23)
		Asian	54.3	0.27	0.786	0.88 (0.36, 2.18)		0.34	0.732	1.41 (0.20, 10.26)
		SCA	0.0	2.84	0.005	0.24 (0.09, 0.64)		2.57	0.010	0.27 (0.10, 0.73)
	*GA* versus *AA*	Overall	63.3	2.46	0.014	1.28 (1.05, 1.56)		1.62	0.104	1.38 (0.94, 2.04)
		Asian	68.1	3.24	0.001	1.49 (1.17, 1.90)		2.02	0.043	1.82 (1.02, 3.26)
		SCA	28.5	0.37	0.709	0.94 (0.67, 1.32)		0.32	0.746	0.94 (0.62, 1.40)
Superdominant	*GA* versus *GG/AA*	Overall	59.9	2.80	0.005	1.32 (1.09, 1.61)		1.84	0.065	1.42 (0.98, 2.06)
		Asian	67.1	3.26	0.001	1.50 (1.17, 1.91)		2.04	0.042	1.81 (1.02, 3.21)
		SCA	17.6	0.21	0.833	1.04 (0.74, 1.45)		0.19	0.850	1.04 (0.71, 1.51)
